# 

**Published:** 1855-07-01

**Authors:** 


					JEo (Eorrcsponticnts.
The conclusion of the articlc on Oinomania is unavoidably postponed until
our next number.

				

